# A comparative study of silver nanoparticle dissolution under physiological conditions†

**DOI:** 10.1039/d0na00733a

**Published:** 2020-10-20

**Authors:** Lukas Steinmetz, Christoph Geers, Sandor Balog, Mathias Bonmarin, Laura Rodriguez-Lorenzo, Patricia Taladriz-Blanco, Barbara Rothen-Rutishauser, Alke Petri-Fink

**Affiliations:** Adolphe Merkle Institute, University of Fribourg Chemin des Verdiers 4 1700 Fribourg Switzerland alke.fink@unifr.ch patricia.taladrizblanco@unifr.ch; International Iberian Nanotechnology Laboratory (INL), Water Quality Group Av. Mestre José Veiga s/n 4715-330 Braga Portugal; School of Engineering, Zurich University of Applied Sciences Technikumstrasse 9 8400 Winterthur Switzerland; Chemistry Department, University of Fribourg Chemin du Musée 9 1700 Fribourg Switzerland

## Abstract

Upon dissolution of silver nanoparticles, silver ions are released into the environment, which are known to induce adverse effects. However, since dissolution studies are predominantly performed in water and/or at room temperature, the effects of biological media and physiologically relevant temperature on the dissolution rate are not considered. Here, we investigate silver nanoparticle dissolution trends based on their plasmonic properties under biologically relevant conditions, *i.e.* in biological media at 37 °C over a period of 24 h. The studied nanoparticles, surface-functionalized with polyvinylpyrrolidone, beta-cyclodextrin/polyvinylpyrrolidone, and starch/polyvinylpyrrolidone, were analysed by UV-Vis spectroscopy, lock-in thermography and depolarized dynamic light scattering to evaluate the influence of these coatings on silver nanoparticle dissolution. Transmission electron microscopy was employed to visualize the reduction of the nanoparticle core diameters. Consequently, the advantages and limitations of these analytical techniques are discussed. To assess the effects of temperature on the degree of dissolution, the results of experiments performed at biological temperature were compared to those obtained at room temperature. Dissolution is often enhanced at elevated temperatures, but has to be determined individually for every specific condition. Furthermore, we evaluated potential nanoparticle aggregation. Our results highlight that additional surface coatings do not necessarily hinder the dissolution or aggregation of silver nanoparticles.

## Abstract 2

The dissolution and aggregation behaviour of silver nanoparticles under physiological conditions and in biologically relevant environments is investigated by exploiting their plasmonic properties.

## Graphical abstract

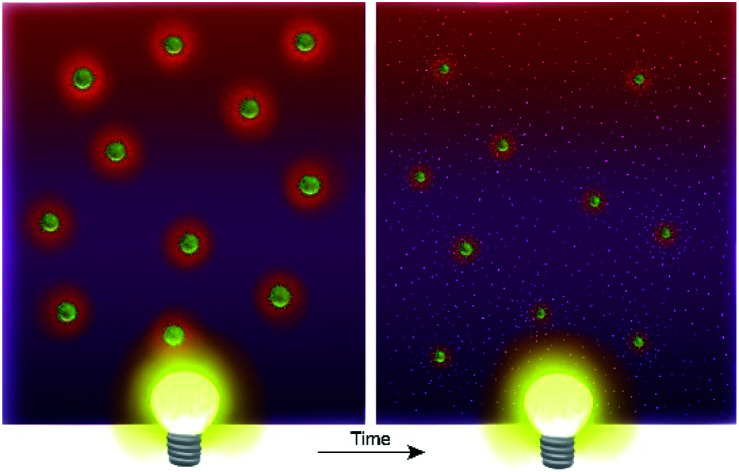

## 1 Introduction

Silver nanoparticle (AgNP) dissolution has been intensively studied in recent years.^1–4^ Its importance arises from the antimicrobial properties of AgNPs and their consequent utilization in a vast range of consumer products, such as textiles, cosmetics, and food packaging.^5–7^ Given that the prominent antibacterial effects of AgNPs originate from the release of silver ions (Ag^+^) upon particle dissolution,^3,8^ the possible adverse effects should be very well understood to ensure maximum safety.

The release of Ag^+^ ions into the environment poses a threat not only to bacteria but also to organisms destined for human consumption in aquatic habitats such as fish, molluscs and seaweed.^9^ It has been shown that bioaccumulated silver is toxic to zebrafish embryos even at concentrations as low as 10 ng mL^−1^.^10^ At cell level, the generation of reactive oxygen species due to the presence of Ag^+^ ions has also been reported.^11^ Furthermore, AgNPs can promote adverse effects such as cytotoxicity by either releasing Ag^+^ ions in solution or entering the cell and subsequently dissolving, which is referred to as the Trojan horse mechanism.^12–17^ Therefore, a number of studies have analysed potential inhibition of Ag^+^ ion release from NPs and reported that the presence of species such as cysteine, glucose or natural organic matter in the system can inhibit AgNP dissolution.^2,18^ It was also observed that reduced temperatures and high pH diminish the release of Ag^+^ ions.^9^ Consequently, it was found that the rate of AgNP dissolution depends on the environmental conditions, but also strongly on their physicochemical properties, such as particle size, shape, crystallinity and surface functionalization.^9,14,15,19,20^ Eventually, the rate decreases with ongoing ion release from the NP surface, as the overall surface area of the system is constantly reducing.^3^ Hence, smaller NPs dissolve faster than bigger ones.^14,15^

The influence of surface functionalization has lately also been the focus of a number of studies. For example, higher ion release has been reported for polyvinylpyrrolidone (PVP)-functionalized AgNPs relative to citric acid (CA)-capped NPs, as Ag^+^ ions are likely to bind to the carboxylate groups of citrate, resulting in reduced solubility.^9,15^ It has also been shown that the antimicrobial properties of AgNPs are reduced upon aggregation,^21–23^ and therefore, surface functionalization contributes to the retention of these antibacterial effects by enhancing the electrostatic and/or steric repulsion between the AgNPs.^20^ NP aggregation results in an overall reduced surface area and therefore leads to diminished ion release rates.^14^ In contrast to NP size, shape and functionalization, the NP concentration has presumably less influence on ion release rates, as Kittler *et al.* reported that the degree of AgNP dissolution seems to be an intrinsic property and does not depend on the absolute NP concentration.^19^

A two-step surface reaction has been proposed by Adamczyk *et al.* to address the oxidative dissolution of AgNPs, contrasting with previously published studies that assumed dissolution to be a single-step process.^3^ The two-step proposition is comprised of an initial Ag_2_O-phase formation on the surface of the NPs followed by acidic dissolution, which ultimately leads to the release of Ag^+^ ions into the environment. Hence, dissolved oxygen is the main oxidizing agent for AgNP dissolution but is not solely responsible for dissolution.^2^ In the case of AgNP oxidation, peroxide intermediates, including H_2_O_2_, are produced, which may act as reactive oxygen species and thus contribute to the toxicity of AgNPs.^9^

Since the toxicity of AgNPs is still under debate, detailed studies of their dissolution behaviour in biological environments are of particular interest for the bionanotechnology community. Hence, we focus on this aspect by making use of the plasmonic properties of AgNPs. For this purpose, we use UV-Vis spectroscopy (UV-Vis), depolarized dynamic light scattering (DDLS), transmission electron microscopy (TEM), and lock-in thermography (LIT). While AgNP dissolution has previously been investigated by exploiting the light absorbance and scattering behaviour of AgNPs (*i.e.* by UV-Vis and DLS),^1,3,20,24^ LIT is yet to be applied in such a context. LIT is a sensitive infrared imaging technique, which measures the thermal energy generated by plasmonic NPs upon excitation with light of specific wavelengths and is able to detect AgNPs in complex environments.^25,26^ As reported in the literature, AgNP dissolution causes the overall absorbance to gradually reduce,^1^ hence by irradiating AgNPs at their plasmon resonance, dissolution can be tracked by changes in the generated heat.

Typically, each of the above-mentioned methods has distinct advantages, but also faces detrimental limitations. We intend to overcome potential obstacles by combining four different analytical techniques to investigate the influence of biological media and temperature on AgNP dissolution. *In vitro* studies are usually performed at 37 °C,^27–29^ which includes incubation of the investigated NPs at this temperature. However, basic NP characterisation, such as NP size and morphology, as well as colloidal stability, is often performed at room temperature (RT) and in water, which neglects the potential influence of elevated temperatures on the dissolution rate of AgNPs. Most commonly, ion release rates are evaluated, which offers the distinct advantage that dissolution can be quantitatively assessed, for instance by inductively-coupled plasma (ICP) analysis.^7,24,30^ However, by following such an approach, dissolution rates might be underestimated due to the adsorption of Ag^+^ ions to dialysis membranes or in the presence of chlorides, which rapidly bind Ag^+^ ions after their release, resulting in the formation of AgCl(s).^1,2^ Here, we evaluate the dissolution kinetics of NPs with three different surface functionalizations, *i.e.* (1) PVP, (2) β-cyclodextrin/PVP (β-CD) and (3) starch/PVP at RT and 37 °C. PVP was chosen as a base functionalization, as it is commonly used as a capping agent of AgNPs in both research and industrial applications.^5,31,32^ Additional surface coatings were added to NP types (2) and (3) to investigate potential reduction of dissolution due to the supplementary capping agents, as varying AgNP dissolution rates have been reported depending on the applied surface functionalization (*e.g.* PVP, CA or polyethylene glycol (PEG)).^1,15,23^

## 2 Experimental section

### 2.1 Chemical and materials

Silver nitrate (99%, AgNO_3_), sodium citrate tribasic dihydrate (≥98%, C_6_H_5_Na_3_O_7_·2H_2_O), tannic acid (99%, C_76_H_52_O_46_), polyvinylpyrrolidone (PVP 10 kDa and 40 kDa, (C_6_H_9_NO)_*n*_), citric acid (CA 99.5%, C_6_H_8_O_7_), d-(+)-glucose (99.5%, C_6_H_12_O_6_), nitric acid (70%, HNO_3_) and β-cyclodextrin (≥97%, C_42_H_70_O_35_) were purchased from Sigma-Aldrich, Switzerland. Sodium hydroxide (≥98%, NaOH) was acquired from Fluka, Switzerland, PVP 8 kDa from Alfa Aesar, Switzerland, and starch ((C_6_H_10_O_5_)_*n*_) from Acros Organics, Switzerland. Phosphate buffered saline (PBS 1×) and Roswell Park Memorial Institute 1640 Medium (cRPMI), supplemented with fetal bovine serum (FBS, 10%, Chemie Brunschwig AG, Switzerland) were purchased from Gibco, Switzerland. Artificial lysosomal fluid (ALF) was prepared as a solution containing 54.93 mM sodium chloride (≥99%, NaCl), 150.01 mM sodium hydroxide (≥98%, NaOH), 108.27 mM citric acid (99.5%, C_6_H_8_O_7_), 0.87 mM calcium chloride (≥97%, CaCl_2_), 0.52 mM magnesium chloride (≥98%, MgCl_2_), 0.67 mM sodium phosphate dibasic (≥99%, Na_2_HPO_4_), 0.27 mM sodium sulphate (≥99%, Na_2_SO_4_), 0.36 mM sodium citrate tribasic dihydrate (≥99%, C_6_H_5_Na_3_O_7_·2H_2_O), 0.39 mM sodium tartrate (≥99%, C_4_H_4_O_6_Na_2_), 0.76 mM sodium l-lactate (98%, C_3_H_5_NaO_3_), 0.78 mM sodium pyruvate (≥99%, C_3_H_3_NaO_3_), 0.64 mM glycerine (98.5%, C_3_H_8_O_3_) and 0.1% formaldehyde (37 wt%, CH_2_O) (except for sodium hydroxide all of these components were purchased from Sigma-Aldrich, Switzerland). Milli-Q water was used for all synthesis and experiments.

### 2.2 Polyvinylpyrrolidone surface-functionalized silver nanospheres (AgNPs@PVP)

Nine mg of silver nitrate were dissolved in 40 mL of Milli-Q water and heated to 60 °C in a 100 mL round-bottomed flask provided with a reflux system. Simultaneously, 25 mg of tannic acid were mixed with 36 mg sodium citrate tribasic dissolved in 10 mL of Milli-Q water and heated to 60 °C. After 5 min both solutions were mixed and brought to boiling under magnetic stirring for 20 min. The resulting approximately 30 nm AgNPs were centrifuged for 25 min at 3500 rpm after cooling down to RT and redispersed in 25 mL of Milli-Q water. The AgNP dispersion was subsequently added dropwise to a 40 mL solution of 0.15 mM PVP (10 kDa) and stirred overnight. The excess PVP was removed by centrifugation for 25 min at 3500 rpm. AgNPs were concentrated in Milli-Q water to a final Ag^0^ concentration of 2 mM (216 μg mL^−1^).

### 2.3 Polyvinylpyrrolidone/β-cyclodextrin-functionalized silver nanospheres (AgNPs@β-CD/PVP)

The synthesis of AgNPs@β-CD/PVP was carried out following the two-step preparation method as described in the literature.^33^ Briefly, β-CD-coated AgNPs were synthesised first, followed by functionalisation with 8 kDa PVP. Therefore, a 60 mL mixture consisting of 15 mL mM d-(+)-glucose (aq., 13 mM), 15 mL NaOH (aq., 10 mM) and 30 mL of β-CD (aq., 15 mM) was heated to 60 °C under stirring. At the desired temperature 20 mL of AgNO_3_ (aq., 10 mM) was added dropwise under vigorous stirring. The formed AgNPs were subsequently dialyzed against Milli-Q water to remove excess reactants. In the second step, 11 mg of PVP were dissolved in Milli-Q water and dropwise added to a total of 40 mL of AgNP@β-CD dispersion. Afterwards, the resulting AgNPs@β-CD/PVP were kept in a shaker for 4 h and finally stored at 4 °C until use.

### 2.4 Polyvinylpyrrolidone/starch-functionalized silver nanospheres (AgNPs@starch/PVP)

20 mL of an aqueous solution of starch was sonicated for 15 min. Afterwards, 4.32 mL of silver nitrate (3.69 mM), 506 μL of d-(+)-glucose (94 mM) and 300 μL of sodium hydroxide (1 M) were added to the starch solution. The mixture was sonicated for one hour, resulting in the formation of AgNPs. Subsequently, the dispersion was cleaned by centrifugation for 40 min at 9000 rpm. AgNPs were redispersed in 23 mL of Milli-Q water and coated with PVP by dropwise addition of 6.38 mL of 40 kDa PVP (10 mg mL^−1^, 63.8 mg of aq. PVP). The mixture was stirred at RT overnight and finally centrifuged twice at 4000 rpm for 30 min.

### 2.5 Sample treatment

For the investigation of AgNP dissolution, samples were prepared as follows: stock dispersions of the three individual AgNPs were diluted in different biologically relevant environments (volume ratio AgNPs to biological media = 1 : 9), cRPMI, PBS 1×, ALF 1×, CA 0.1 M and Milli-Q water as control, to obtain a final concentration of Ag^0^ = 0.2 mM (21.6 μg mL^−1^). Before the experiments, samples were prepared as aliquots for the individual time points and either kept at RT or incubated at 37 °C.

### 2.6 Lock-in thermography (LIT)

To analyse AgNP dissolution, LIT measurements were performed using a custom-made setup operating as reported previously.^26^ Briefly, a multi-wavelength LED-based light source (AN178_2 61 LED module, ADOM, Germany) was used to stimulate the AgNPs at specific wavelengths and an infrared camera (Onca-MWIR-InSb-320, XenICs, Belgium) mounted on a standard microscope stand (Leica Microsystems, Germany) recorded the thermal signals originating from the investigated NP dispersions. The camera contains an InSb array (320 × 256 pixels), which operates in the mid-IR range (3 to 5 μm) and is capable of capturing full-frame images at a rate of up to 200 Hz. A camera-link frame grabber transfers the acquired infrared images to a computer where they are processed in real time by a custom-developed LabVIEW software according to the digital lock-in principle.^25,34^ The results are 2D amplitude maps (in kelvin) that are proportional to the sample generated heat. A 40 μL dispersion of AgNPs in water or biological media was excited with a source of light centred at 400 nm and 660 nm, with power densities of 58 mW cm^−2^ and 136 mW cm^−2^, respectively. Samples were measured five times for 60 s each to ensure reproducibility. Homogeneous illumination of the dispersions was ensured by placing a light-homogenizing glass rod (N-BK7, Edmond Optics, USA) directly between the LED module and the sample holder. The modulation frequency was set to 1 Hz. Custom-made, half-spherical and light-transparent sample holders made from polystyrene were used for all NP dispersions. The resulting amplitude images were analysed (ImageJ v1.52b, NIH, USA & Origin 2016, OriginLab, USA) to extract signal mean over the sample area and standard deviation values.

### 2.7 UV-Vis spectroscopy (UV-Vis)

UV-Vis spectra of AgNPs in water and biological media were recorded at 37 °C and RT with a V-670 spectrophotometer (Jasco, USA) using a sealed 10 mm path length quartz suprasil cuvette (Hellma Analytics, Germany). For the 24 h UV-Vis measurements, samples were measured continuously and spectra were taken every hour. The background of the investigated biological media was subtracted from the UV-Vis spectra.

### 2.8 Transmission electron microscopy (TEM)

A Tecnai Spirit transmission electron microscope (FEI, USA) operating at 120 kV was used to evaluate the AgNP diameter before and after the sample treatment. TEM grids were prepared as follows: 10 μL dispersions of AgNPs were dropcast on parafilm, and a copper carbonated TEM grid was placed on top of the drop for 10 min. Subsequently, the grids were rapidly dried by spinning them in a spin coater (WS-650MZ, Laurell, USA) at 2000 rpm for 3 min. TEM images were acquired with a Veleta CCD camera (Olympus, Japan) and were processed with ImageJ (v1.52).

### 2.9 Depolarized dynamic light scattering (DDLS)

Light scattering data were collected at constant temperature (37 °C) using a commercial goniometer instrument (3D LS Spectrometer, LS Instruments AG, Switzerland). The primary beam was formed by a linearly polarized and collimated laser beam (Cobolt 05-01 diode pumped solid state laser, *λ* = 660 nm, *P* max. = 500 mW, Hübner Photonics, Germany), and the scattered light was collected by single-mode optical fibres equipped with integrated collimation optics. Glass tubes (10 × 75 mm, Kimble, Germany) were filled with 2 mL dispersions of AgNPs and measured at 37 °C. The scattering angle was set to 30° and the laser intensity to 80 kHz. Measurements were performed over 24 h, consisting of consecutive runs of 60 s. Cumulant fitting of the DDLS data sets was performed using Wolfram Mathematica (version 11.2) and according to published routines.^35–37^

### 2.10 Inductively-coupled plasma optical emission spectrometry (ICP-OES)

ICP-OES (PerkinElmer Avio-200, USA, axial-viewing, *λ* = 328.068 nm) was used to determine the concentration of the investigated AgNPs. 0.5 mL of an AgNP dispersion (Ag ∼ 0.1 mM) was dissolved in 1.38 mL of HNO_3_ overnight at RT and further diluted with 8 mL of Milli-Q water. Six Ag standards (Sigma-Aldrich, Switzerland) and one blank were prepared following the same protocol. Subsequently, ICP-OES analysis was carried out using a radio frequency power of 1500 W, gas flow rates of 8 L min^−1^ (Ar, plasma) and 0.2 L min^−1^ (N_2_, auxiliary), pump of 0.7 L min^−1^ (nebulizer), and a 1 mL min^−1^ sample flow rate (equilibration delay of 90 s). Samples were prepared and measured in triplicate.

### 2.11 pH measurements

The pH values of the AgNP dispersions were determined with a sympHony meter equipped with a pH SM 123 electrode (VWR, USA).

## 3 Results and discussion

An advantage of investigating the changes in AgNP absorbance upon dissolution compared to Ag^+^ ion release rates lies in the redundant separation of NPs and ions, *e.g.* by dialysis. Hence, previous studies have focussed primarily on the use of UV-Vis to investigate the absorbance of AgNPs *in situ* while undergoing dissolution.^1,20,30^

**Fig. 1:**
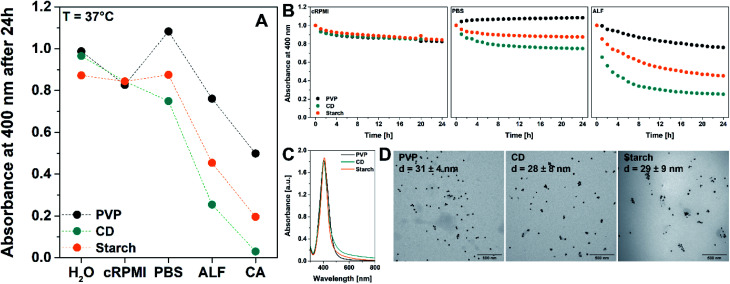
(A) Dissolution of 30 nm AgNPs coated with PVP only (black dots), β-CD/PVP (CD, green dots), and starch/PVP (starch, orange dots) in water, cRPMI, PBS 1×, ALF 1× and 0.1 M CA at 37 °C after 24 h of incubation. Values were subtracted from the UV-Vis trends normalized to *t*_0_. (B) UV-Vis dissolution trends of AgNPs coated with PVP only (black dots), β-CD/PVP (CD, green dots), and starch/PVP (starch, orange dots) over 24 h at 37 °C in cRPMI, PBS 1× and ALF 1×. Measurements were carried out by recording the absorbance at 400 nm in one-hour intervals and normalising to *t*_0_. The corresponding results for water and CA are shown in Fig. S2.† (C) UV-Vis spectra of the synthesised AgNPs and TEM micrographs (D). Scale bar: 500 nm. The diameter (*d*) represented in the images corresponds to the core diameter of the NPs. An enlarged view of the TEM images including histograms can be found in the ESI (Fig. S1†).

In the context of this work, 30 nm surface-coated AgNPs with three different coatings – PVP, β-CD/PVP and starch/PVP – were synthesised (Fig. 1C and D) and analysed at physiological temperature (37 °C) over 24 h using UV-Vis, LIT, DDLS and TEM. For simplicity, we will refer to these NPs as PVP, CD and starch, respectively. These different coatings are expected to alter the AgNP dissolution rates as: (a) the diffusion of the released Ag^+^ ions is potentially slowed down due to the increased molecular weight of the coating, and (b) protein corona formation is affected by the different coatings.^1^ The latter is also known to influence NP dissolution rates.^38^

We dispersed the AgNPs in biological media that are commonly used for *in vitro* studies, namely complete Roswell Park Memorial Institute 1640 cell culture medium (cRPMI, pH 7.9), phosphate-buffered saline (PBS 1×, pH 7.3) and artificial lysosomal fluid (ALF 1×, pH 4.1). 0.1 M citric acid solution (CA, pH 2.0) was employed to evaluate the effect of low pH on AgNP dissolution, and Milli-Q water (pH 7.0) was used as a control. Results were compared to those obtained at RT.

Fig. 1A summarises the effects of the applied coatings on AgNP dissolution in water, cRPMI, PBS 1×, ALF 1×, and CA, as obtained by UV-Vis at 37 °C and after incubation for 24 h. Negligible dissolution was observed for AgNPs coated with PVP and CD in water (1.3%, 3.5%, respectively), whereas starch NPs dissolve to a small degree (12.8%) (Table 1). In contrast to dispersions of AgNPs in water, a low dissolution rate of AgNPs in cRPMI and PBS 1× was observed over 24 h (Fig. 1B, S1 and S2†), whereas in ALF 1× and CA the aforementioned chemical nature of media significantly enhances the dissolution of the AgNPs. The decrease in absorbance for NPs dispersed in cRPMI suggests that proteins present in the media and the resulting protein corona do not sufficiently stabilize the NPs.^38,39^ Interestingly, starch NPs dissolve at similar rates in cRPMI and water at 37 °C. Starch and PVP NPs also present similar trends in cRPMI at 37 °C. In PBS 1× after 24 h, CD NPs display highest dissolution (25.0%), followed by starch NPs (12.5%). PVP NPs exhibit a rise in absorbance by 8.3% in PBS 1×, which is likely explained by slight variation of PVP conformation on the AgNP surface, which can modify the refractive index and the thickness of the coating.^40^ Independent of the coating, NPs drastically dissolve in ALF 1× and CA, and we assume that this effect is caused by the low pH (pH_ALF_ = 4.1, pH_CA_ = 2.0), and the high ionic strength (*I*_ALF_ = 0.34 mol L^−1^,^24^*I*_CA_ = 0.0034 mol L^−1^) of the media.^9,18^ AgNPs functionalized with CD exhibit the highest dissolution rates of all three investigated NP types in PBS 1×, ALF 1× and CA at 37 °C after 24 h (Fig. 1), which indicates that these NPs are more susceptible to dissolve than those functionalized with PVP and starch (Table 1). Our results show that coating the NPs with only PVP is more effective against dissolution than using additional CD or starch layers.

**Table 1:** Percentage of AgNP dissolution in water, cRPMI, PBS 1×, ALF 1× and 0.1 M CA after 24 h of incubation. Values were extracted from the normalised UV-Vis spectra and calculated according to the formula: 100(Abs_*t*=24 h_ − Abs_*t*=0 h_). Negative values correspond to increments in absorbance over time, which is attributed to conformational changes of the PVP on the NP surface that modify the refractive index and thickness of the coating, thereby leading to alterations of the plasmon resonance conditions^40^

		% dissolution at 24 h obtained by UV-Vis				
		H_2_O	cRPMI	PBS	ALF	CA
37 °C	PVP	1.3	17.3	−8.3	23.9	50.1
	CD	3.5	15.6	25.0	74.6	97.0
	Starch	12.8	15.6	12.5	54.6	80.4
RT	PVP	−1.5	4.6	66.6	63.5	67.9
	CD	0.2	9.0	34.7	46.7	41.5
	Starch	4.3	4.6	6.8	22.3	47.5

As indicated by the absorbance spectra, aggregation of CD and starch NPs is present in ALF 1× and distinct for CA (Fig. S4–S6†), while NPs coated only with PVP remain stable in both media. In contrast, NP aggregation is not observed in water, cRPMI or PBS 1× at 37 °C. It is known that CA cross-links starch by hydrogen bonding,^41^ which can explain the aggregation of starch NPs dispersed in CA (both at 37 °C and RT). A similar mechanism is proposed for the CA-induced aggregation of AgNPs functionalized with CD, as cross-linking of β-CD by CA has been reported in the literature.^42^ Aggregation of NPs dispersed in ALF is likely caused by the high ionic strength and the fact that CA is one of the main components of ALF, and therefore present in the solution.^24^

**Fig. 2:**
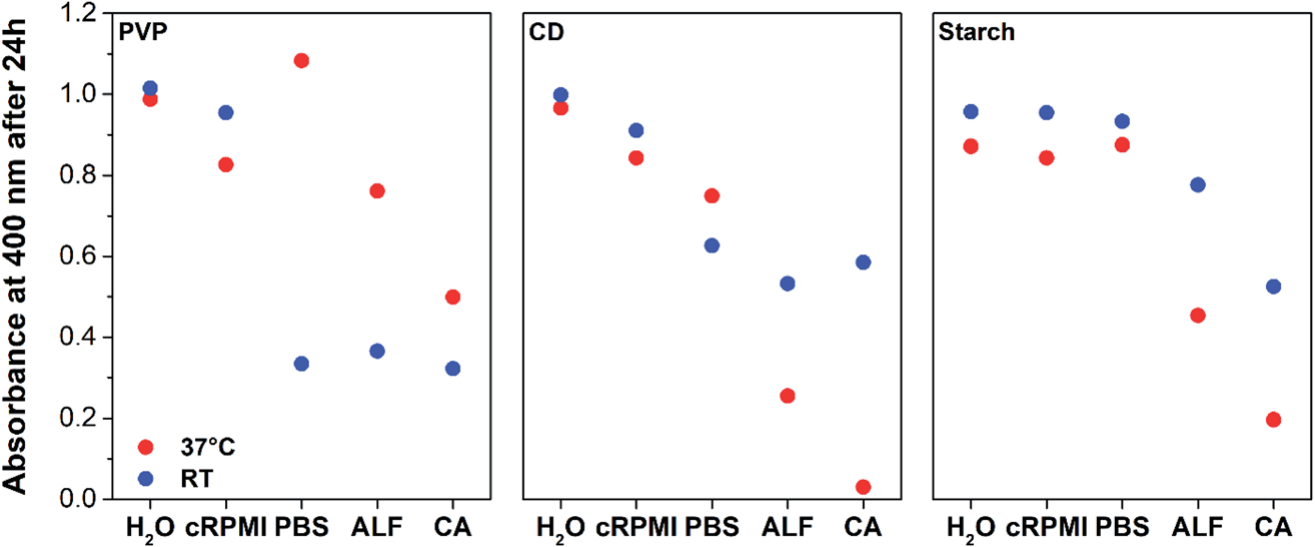
Temperature effect on AgNPs dissolution in water, cRPMI, PBS 1×, ALF 1× and 0.1 M CA after 24 h of incubation, as recorded by UV-Vis. Red and blue dots represent data obtained at 37 °C and RT, respectively.

All NPs except those coated with PVP in PBS 1×, ALF 1× and CA dissolve slower at RT than at 37 °C, with the starch NPs being the least affected by temperature changes (Fig. 2, S2† and Table 1). In contrast to the observations at 37 °C, NPs coated only with PVP show the highest dissolution at RT when compared to those coated with CD and starch in PBS 1× and ALF 1× at the same temperature (Fig. S7† and Table 1), indicating that the stability of PVP is compromised under these conditions. In ALF 1× at RT, the NPs dissolved to a similar extent independent of the coatings, whereas at 37 °C a significant influence on AgNP dissolution is evident. Dissolution of the NPs coated with CD in CA at RT is not as drastic as that observed at 37 °C. After 24 h only 41.5% of the NPs were dissolved at RT compared to 97.0% at 37 °C (Table 1). Similarly, enhanced dissolution is observed for starch NPs in CA at 37 °C (80.4%) with respect to RT (47.5%). These results confirm the massive influence of temperature on AgNP dissolution and highlight the necessity to perform these studies at a physiological temperature, especially if the final goal is to apply the NPs to *in vivo* or *in vitro* studies. The reasons for the enhanced dissolution behaviour of AgNPs coated with PVP in PBS 1×, ALF 1× and CA at RT remain unclear.

**Fig. 3:**
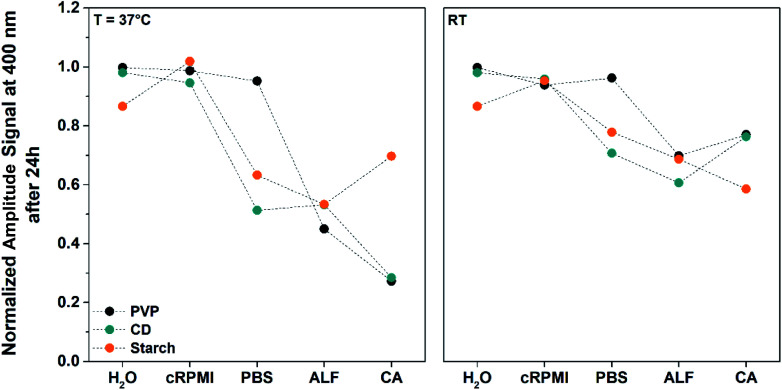
Dissolution of 30 nm AgNPs coated with PVP only (black dots), β-CD/PVP (CD, green dots) and starch/PVP (starch, orange dots) measured by LIT at 37 °C and RT over 24 h in water, cRPMI, PBS 1×, ALF 1× and 0.1 M CA using a source of light centred at 400 nm. Values were subtracted from the LIT amplitude signals normalized to the *t*_0_ value of every individual condition (Fig. S8 and S9†). Time points were measured every 45 min up to 6 h and then subsequently after 8, 12 and 24 h.

During AgNP dissolution the overall surface area of the NPs is reduced, leading to a reduction of their plasmonic heating efficiency.^43^ Hence, it is possible to study AgNP dissolution in biological media by evaluating their characteristic heating behaviour measured by LIT at 37 °C and RT over 24 h. LIT can resolve temperature differences down to 0.001 K for AuNPs and superparamagnetic iron oxide NPs, and is therefore likewise sensitive enough to changes in the maximum light absorbance of AgNPs caused by dissolution effects.^1,25^ To properly compare our findings, we kept the AgNP concentration constant at 0.2 mM (21.6 μg mL^−1^) for all experiments and normalized our LIT amplitude signals with *t*_0_ of every individual condition (Fig. 3, S7 and S8†).

As previously reported, depending on the pH, temperature, NP surface functionalization and solvent, AgNP dissolution and aggregation can occur simultaneously.^1,20^ In order to investigate the simultaneous dissolution and aggregation in the media mentioned above, we measured the samples at excitation wavelengths of 400 nm and 660 nm (Fig. S8 and S9†). Aggregation of AgNPs induces red-shifts in absorbance, resulting in heat generation at higher wavelengths.^26^ In this context, NP aggregation is expected to lead to pronounced heat signals at 660 nm, whereas dissolution is directly observed by decreasing heat signals at 400 nm. The media do not directly contribute to the heat generation, except for cRPMI and in particular its phenol red component, which absorbs light from the UV region up to *ca.* 600 nm (Fig. S10†). However, this heating contribution is stable over 24 h and can therefore be neglected.

Fig. 3 shows the LIT results of the three investigated AgNPs dispersed in cRPMI, PBS 1×, ALF 1×, CA, and water, at 37 °C and RT after incubation for 24 h. In water and cRPMI (except for starch NPs in water), LIT displays constant amplitude signals (*i.e.* generated heat), indicating that the NPs are stable under these conditions. Aggregation of NPs can also be excluded, as no notable heat generation at 660 nm is observed with LIT (Fig. S9†). In contrast to water and cRPMI, AgNPs exhibit a decrease in amplitude signal over time when dispersed in PBS 1×, ALF 1× and CA, except for PVP NPs in PBS 1×, an effect that was also reported to occur at RT by Loza *et al.*^2^ This decrease was less pronounced for starch NPs in CA at both 37 °C and RT than for CD and PVP AgNPs (Fig. S8†). Enhanced dissolution was detected at 37 °C with respect to RT for all conditions, with the exception of starch NPs in CA, whereby the NPs dissolve faster at RT (Fig. 3 and S8† and Table 2). LIT results point to CA as the dominant dissolving condition at 37 °C for PVP and CD NPs, generating only 27.3% and 28.4% of the initial heat after 24 h, respectively, in contrast to 45.0% (PVP) and 53.3% (CD) obtained for ALF 1× (Table 2). Interestingly, both CD and PVP NPs dissolve in a comparable range to ALF 1× and CA at 37 °C. Conversely, the dissolution of NPs coated with starch is more dominant in ALF 1× (Heat_24h,37°C_ = 53.3%) than in CA (Heat_24h,37°C_ = 69.7%) (Fig. 3 and Table 2). In PBS 1× at 37 °C, CD NPs dissolve in a comparable range to those in ALF 1×.

**Table 2:** Percentage of heat generated by the AgNP dispersions upon dissolution in water, cRPMI, PBS 1×, ALF 1× and 0.1 M CA after 24 h at RT and 37 °C. Values were subtracted from the normalised LIT dissolution trends (Fig. S8 and S9)

		% of heat produced by the AgNPs at 24 h obtained by LIT				
		H_2_O	cRPMI	PBS	ALF	CA
37 °C	PVP	99.8	98.7	95.2	45.0	27.3
	CD	98.0	94.6	51.3	53.3	28.4
	Starch	86.6	101.9	63.3	53.3	69.7
RT	PVP	99.8	93.8	96.2	69.8	77.0
	CD	98.0	95.9	70.7	60.7	76.3
	Starch	86.6	95.3	77.8	68.7	58.6

At RT, the dissolution behaviour is in direct contrast to that observed at 37 °C, *i.e.* PVP and CD NPs (Heat_24h,RT_ = 69.8% and 60.7%) dissolve slightly more in ALF 1× than in CA (Heat_24h,RT_ = 77.0% and 76.3%) and starch NPs dissolve more effectively in CA than in ALF 1× (Heat_24h,RT_ = 58.6% *vs.* 69.8%). Furthermore, NPs coated with CD in PBS 1× (Heat_24h,RT_ = 70.7%) dissolve in a comparable range to CA (Heat_24h,RT_ = 76.3%), highlighting once again the influence of temperature on AgNP dissolution.

Similarities, but also differences, were observed after analysing the NPs by LIT and UV-Vis. Both LIT (Fig. 3 and Table 2) and UV-Vis (Fig. 1A and Table 1) consistently indicate that CA is the dominant dissolving condition at 37 °C for PVP and CD NPs. Both techniques show stable PVP and CD NPs and a slight dissolution of starch NPs in water at RT and 37 °C. LIT and UV-Vis indicate that all NPs all stable in cRPMI at RT. For cRPMI at 37 °C, LIT shows stable NPs independent of the coating type, whereas UV-Vis indicates slight dissolution of *ca.* 15%. Starch NPs in CA at 37 °C, seem evidently more stable when characterised by LIT than by UV-Vis. With the exception of PVP and CD NPs at RT, LIT measurements indicate greater dissolution for PBS 1× than as measured by UV-Vis. In contrast, enhanced dissolution is predominantly observed for NPs dispersed in ALF 1× and CA by UV-Vis relative to LIT. These findings are supported by a supervised machine learning model based on neural networks, in which all relevant conditions are considered with respect to UV-Vis, LIT and TEM analysis (Fig. S11 and Table S1†). Judging from this model, it can be stated that the surrounding media have the strongest influence on AgNP dissolution, followed by coating and temperature.

The most striking difference between UV-Vis and LIT is the stability of PVP NPs at RT. While these NPs show enhanced dissolution in PBS 1×, ALF 1× and CA at RT by UV-Vis analysis, LIT measurements reveal stronger dissolution at 37 °C. As indicated by the absorbance spectra (Fig. S4–S6†), aggregation of CD and starch NPs is present in ALF 1× and CA, whereas NPs seem to be stable in water, cRPMI and PBS 1×. However, NP aggregation was detected only for CD NPs in CA by LIT, which points out the lack of sensitivity of LIT towards low amounts of aggregates when irradiating the samples at 660 nm (Fig. S9†). Discrepancies between the LIT and UV-Vis techniques observed when measuring at 400 nm can also be attributed to the lower sensitivity of the infrared camera in comparison to UV-Vis, as well as differences in the experimental setup, since samples were measured continuously by UV-Vis while aliquots had to be taken for LIT analysis and these were investigated consecutively.

Although LIT and UV-Vis have some limitations, they offer advantages over other techniques such as DDLS and TEM for AgNP dissolution studies. DDLS provides scattering information originating exclusively from the plasmonic NPs on an essentially zero-background in presence of proteins as reported by Balog *et al.*, which is not achievable using conventional DLS.^44^ Moreover, AgNPs promote higher enhancement of depolarized scattering owing higher scattering contribution in their plasmonic properties.^45,46^ However, due to the formation of small aggregates and the high sensitivity of DDLS towards changes in volume, highly inconsistent hydrodynamic diameters were obtained for all samples measured at 37 °C (data not shown). Therefore, it is necessary to evaluate the scattering intensity, as a function of AgNP dissolution behaviour. A decrease in scattering intensity is related to a reduction of hydrodynamic diameter, and thus indicates AgNP dissolution. As LIT and UV-Vis results, DDLS shows that all three AgNP types are stable in water (Fig. S12A, F and K†). For NPs coated with PVP, no significant decrease in scattering intensity was observed over 24 h, except for ALF 1× (Fig. S12A–E†). Occasionally, and especially for CD and starch NPs in PBS 1×, ALF 1×, and CA, a sharp rise in scattering intensity was observed within the first hour, which indicated the formation of aggregates (Fig. S12H–J and M–O†). Based on our results, the use of DDLS for this study is limited. As mentioned above, only the scattering intensity can be evaluated, and not the hydrodynamic diameter. For conditions in which NP aggregation occurs, spikes in scattering intensity can lead to an oversaturation of the detector, resulting in the abrupt and unintended abortion of a measurement. The polydispersity of the samples, as well as secondary reactions between the Ag^+^ ions, the capping agents, and the biological media, can influence the scattering signal, thus causing misleading results.^47^ In this context, more elaborate approaches would be required to study the AgNP dissolution. Furthermore, it is worth noting that DDLS is not a simple technique, requiring extensive expertise in data analysis.

In contrast to DDLS, TEM analysis allows determination of the core diameter of the AgNPs. In theory, evaluation of NP dissolution by simply comparing the core diameters should be possible. However, due to the high polydispersity of AgNPs, and thus their high standard deviations, small differences in their core diameters (*i.e.* a few nm) caused by dissolution effects become insignificant (Table S2†). In addition, the presence of salts and proteins impedes the correct TEM analysis due to the formation of protein aggregates, fractals or crystals, which exhibit similar contrast than AgNPs.^48^ The evaluation of NP diameter becomes non-trivial when NPs are aggregated or overlap, which is likely induced not only for the initial state of the AgNPs in specific medium (*e.g.* CA or ALF) but also by drying effects or exceedingly high concentrations on the TEM grid (Fig. S13†). Evaluating AgNP aggregation by TEM is feasible, but one has to consider the potential impact of drying artefacts, which are known to alter NP distribution on the TEM grid.^49^

Overall, the analysis of AgNP dissolution by TEM and DDLS is time-consuming and error-prone, highlighting distinct drawbacks when compared to LIT and UV-Vis. In particular, sample preparation and handling, measurement duration, as well as data treatment and interpretation, are demanding for TEM and also to some degree for DDLS, while UV-Vis and LIT stand out due to their straightforward analysis approach.

## 4 Conclusion

In this study, we investigated the dissolution behaviour of surface-functionalized 30 nm AgNPs at 37 °C and RT over 24 h in biological media. We employed three different types of surface coatings, namely PVP, β-CD/PVP and starch/PVP, to evaluate the influence of additional surface capping. Four different techniques, UV-Vis, LIT, DDLS, and TEM were used to analyse the dissolution kinetics. Dissolution was most prominently observed for all AgNPs dispersed in CA, followed by ALF 1×, for both 37 °C and RT. We found that dissolution is generally enhanced at elevated temperatures, with the exception of AgNPs functionalized with PVP. In specific cases, AgNPs dissolved to a small extent in water and protein-enriched cell culture medium, but were mostly stable at both applied temperatures. We also showed that AgNPs aggregated in CA and ALF 1×. In terms of AgNP aggregation, we noticed that LIT suffers from a lower sensitivity than UV-Vis, which is undoubtedly one of the most sensitive methods for the analysis of plasmonic NP systems. A prominent factor for the differences in observations between the analytical methods is likely based on the high polydispersity of AgNPs, which is further enhanced due to dissolution and is known to impact NP absorbance behaviour. Nevertheless, all employed techniques have their limitations, as demonstrated most prominently for TEM and DDLS in this work, and it is therefore recommended to use a combination of analytical methods for the analysis of NP dissolution. A detailed study on the interactions of Ag^+^ ions with the surface functionalizations would be a beneficial future direction, in order to fully understand the diffusion and adsorption mechanisms of the released ions, as well as potential recrystallisation effects.

## Conflicts of interest

Christoph Geers and Mathias Bonmarin both have equity in the company NanoLockin GmbH, which specializes in lock-in thermal imaging instruments for NP analysis and might benefit from potential interest in this work.

## Supplementary Material

NA-002-D0NA00733A-s001

## References

[cit1] Zook J. M., Long S. E., Cleveland D., Geronimo C. L. A., MacCuspie R. I. (2011). Anal. Bioanal. Chem..

[cit2] Loza K., Diendorf J., Sengstock C., Ruiz-Gonzalez L., Gonzalez-Calbet J. M., Vallet-Regi M., Köller M., Epple M. (2014). J. Mater. Chem. B.

[cit3] Adamczyk Z., Oćwieja M., Mrowiec H., Walas S., Lupa D. (2016). J. Colloid Interface Sci..

[cit4] Abram S. L., Fromm K. M. (2020). Chem.–Eur. J..

[cit5] Kaegi R., Voegelin A., Ort C., Sinnet B., Thalmann B., Krismer J., Hagendorfer H., Elumelu M., Mueller E. (2013). Water Res..

[cit6] Mitrano D. M., Rimmele E., Wichser A., Erni R., Height M., Nowack B. (2014). ACS Nano.

[cit7] Echegoyen Y., Nerín C. (2013). Food Chem. Toxicol..

[cit8] Lok C. N., Ho C. M., Chen R., He Q. Y., Yu W. Y., Sun H., Tam P. K. H., Chiu J. F., Che C. M. (2007). J. Biol. Inorg Chem..

[cit9] Liu J., Hurt R. H. (2010). Environ. Sci. Technol..

[cit10] Yeo M. K., Kang M. (2008). Bull. Korean Chem. Soc..

[cit11] Prabhu S., Poulose E. K. (2012). Int. Nano Lett..

[cit12] Lubick N. (2008). Environ. Sci. Technol..

[cit13] Park E. J., Yi J., Kim Y., Choi K., Park K. (2010). Toxicol. In Vitro.

[cit14] Zhang W., Yao Y., Sullivan N., Chen Y. (2011). Environ. Sci. Technol..

[cit15] Dobias J., Bernier-Latmani R. (2013). Environ. Sci. Technol..

[cit16] Chao J. B., Zhou X. X., Shen M. H., Tan Z. Q., Liu R., Yu S. J., Wang X. W., Liu J. F. (2015). Environ. Sci. Technol..

[cit17] Studer A. M., Limbach L. K., Van Duc L., Krumeich F., Athanassiou E. K., Gerber L. C., Moch H., Stark W. J. (2010). Toxicol. Lett..

[cit18] Liu J., Sonshine D. A., Shervani S., Hurt R. H. (2010). ACS Nano.

[cit19] Kittler S., Greulich C., Diendorf J., Köller M., Epple M. (2010). Chem. Mater..

[cit20] Li X., Lenhart J. J., Walker H. W. (2012). Langmuir.

[cit21] Shrivastava S., Bera T., Roy A., Singh G., Ramachandrarao P., Dash D. (2007). Nanotechnology.

[cit22] Teeguarden J. G., Hinderliter P. M., Orr G., Thrall B. D., Pounds J. G. (2007). Toxicol. Sci..

[cit23] Kvítek L., Panáček A., Soukupová J., Kolář M., Večeřová R., Prucek R., Holecová M., Zbořil R. (2008). J. Phys. Chem. C.

[cit24] Stebounova L. V., Guio E., Grassian V. H. (2011). J. Nanoparticle Res..

[cit25] Breitenstein O. , Warta W. and Schubert M. C., Lock-in Thermography, Springer International Publishing, 3rd edn, 2018, vol. 10

[cit26] Steinmetz L., Taladriz-Blanco P., Geers C., Spuch-Calvar M., Bonmarin M., Balog S., Rothen-Rutishauser B., Petri-Fink A. (2019). Part. Part. Syst. Charact..

[cit27] Tu H.-L., Lin Y.-S., Lin H.-Y., Hung Y., Lo L.-W., Chen Y.-F., Mou C.-Y. (2009). Adv. Mater..

[cit28] Kaba S. I., Egorova E. M. (2015). Nanotechnol., Sci. Appl..

[cit29] Chortarea S., Zerimariam F., Barosova H., Septiadi D., Clift M. J. D., Petri-Fink A., Rothen-Rutishauser B. (2019). Appl. In Vitro Toxicol..

[cit30] Sikder M., Lead J. R., Chandler G. T., Baalousha M. (2018). Sci. Total Environ..

[cit31] Koczkur K. M., Mourdikoudis S., Polavarapu L., Skrabalak S. E. (2015). Dalton Trans..

[cit32] Hansen U., Thünemann A. F. (2015). Langmuir.

[cit33] Andrade P. F., de Faria A. F., da Silva D. S., Bonacin J. A., Gonçalves M. do C. (2014). Colloids Surf., B.

[cit34] Monnier C. A., Lattuada M., Burnand D., Crippa F., Martinez-Garcia J. C., Hirt A. M., Rothen-Rutishauser B., Bonmarin M., Petri-Fink A. (2016). Nanoscale.

[cit35] Koppel D. E. (1972). J. Chem. Phys..

[cit36] Frisken B. J. (2001). Appl. Opt..

[cit37] Geers C., Rodriguez-Lorenzo L., Urban D. A., Kinnear C., Petri-Fink A., Balog S. (2016). Nanoscale.

[cit38] Urban D. A., Rodriguez-Lorenzo L., Balog S., Kinnear C., Rothen-Rutishauser B., Petri-Fink A. (2016). Colloids Surf., B.

[cit39] Lynch I., Dawson K. A. (2008). Nano Today.

[cit40] Jatschka J., Dathe A., Csáki A., Fritzsche W., Stranik O. (2016). Sens. Bio-Sens. Res..

[cit41] Reddy N., Yang Y. (2010). Food Chem..

[cit42] Junthip J. (2019). J. Macromol. Sci., Part A: Pure Appl. Chem..

[cit43] Baffou G., Quidant R. (2013). Laser Photonics Rev..

[cit44] Balog S., Rodriguez-Lorenzo L., Monnier C. A., Obiols-Rabasa M., Rothen-Rutishauser B., Schurtenberger P., Petri-Fink A. (2015). Nanoscale.

[cit45] Balog S., Rodriguez-Lorenzo L., Monnier C. A., Michen B., Obiols-Rabasa M., Casal-Dujat L., Rothen-Rutishauser B., Petri-Fink A., Schurtenberger P. (2014). J. Phys. Chem. C.

[cit46] Israelsen N. D., Hanson C., Vargis E. (2015). Sci. World J..

[cit47] Prucek R., Panáček A., Fargašová A., Ranc V., Mašek V., Kvítek L., Zbořil R. (2011). CrystEngComm.

[cit48] Franken L. E., Boekema E. J., Stuart M. C. A. (2017). Adv. Sci..

[cit49] Michen B., Geers C., Vanhecke D., Endes C., Rothen-Rutishauser B., Balog S., Petri-Fink A. (2015). Sci. Rep..

