# Supplementary material for: A comparative study of silver nanoparticle dissolution under physiological conditions
Source: Nanoscale Adv. 2020 Oct 20;2(12):5760–8. doi: 10.1039/d0na00733a (PMC9417474; doi:10.1039/d0na00733a)
Supplement: NA-002-D0NA00733A-s001 [file NA-002-D0NA00733A-s001.pdf]

Electronic Supplementary Material (ESI) for Nanoscale Advances.
This journal is © The Royal Society of Chemistry 2020

Supporting Information

A comparative study of silver nanoparticle dissolution under physiological conditions

Lukas Steinmetz, Christoph Geers, Sandor Balog, Mathias Bonmarin, Laura Rodriguez-Lorenzo, Patricia Taladriz-Blanco, Barbara Rothen-Rutishauser, Alke Petri-Fink**

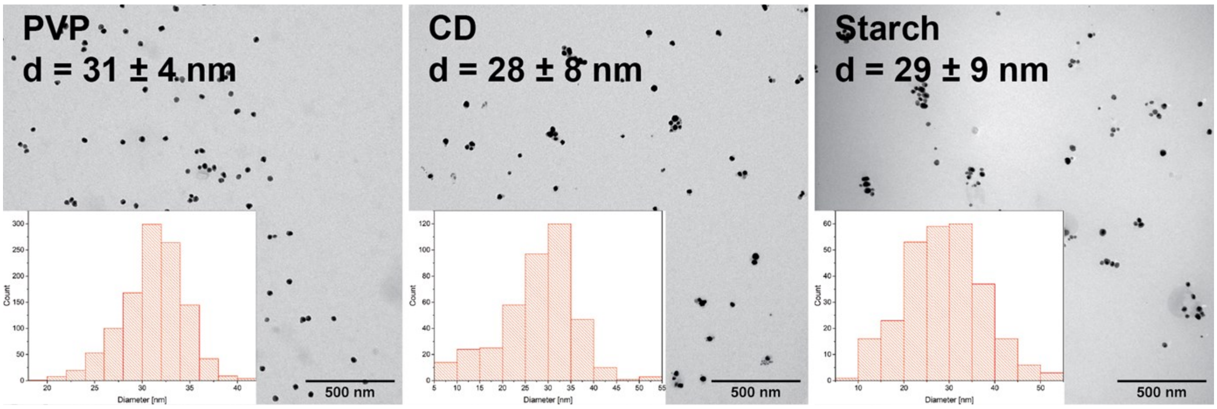

Figure S1. TEM images and corresponding histograms of freshly prepared 30 nm AgNPs coated with PVP only, β-CD/PVP (CD), and starch/PVP (Starch) in Milli-Q water.

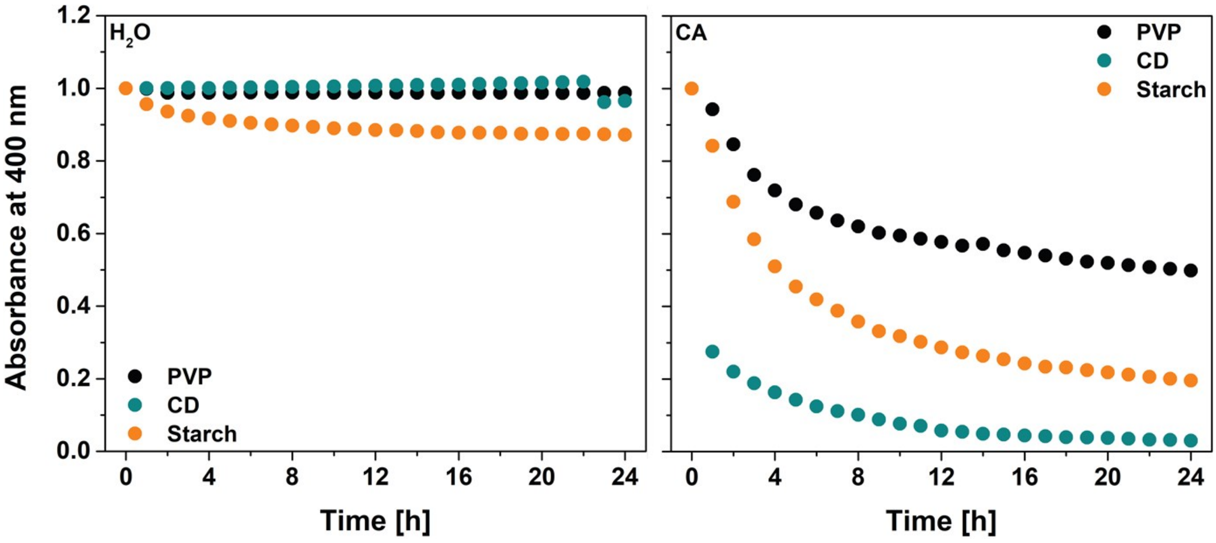

Figure S2. UV-Vis dissolution trends of AgNPs coated with PVP only (black dots), β-CD/PVP (CD, green dots), and starch/PVP (starch, orange dots) over 24 hours at 37 °C in water (left) and citric acid (CA) (right). Measurements were carried out by recording the absorbance at 400 nm in 1 h intervals and normalising it to t_0.

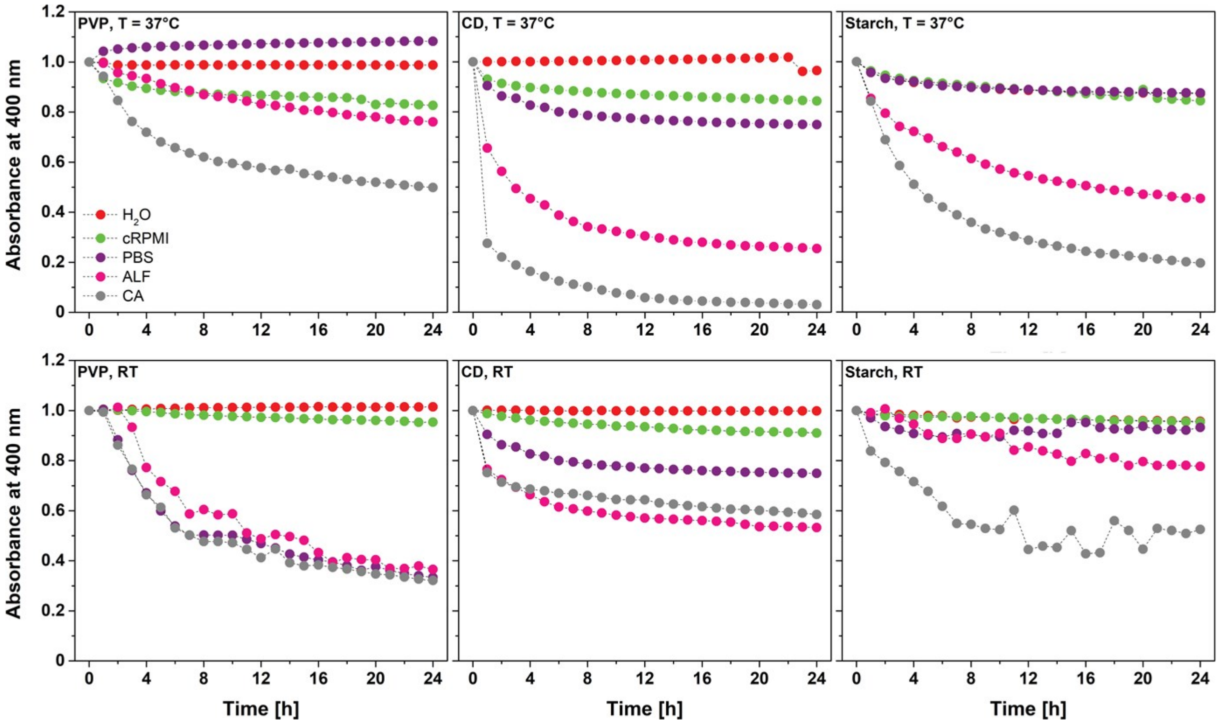

Figure S3. UV-Vis dissolution trends of AgNPs coated with PVP only, β-CD/PVP (CD), and starch/PVP (starch) over 24 hours at 37 °C and RT in water (red dots), cRPMI (green dots), PBS1x (purple dots), ALF1x (pink dots) and citric acid (CA) (grey dots). Measurements were carried out by recording the absorbance at 400 nm in 1 h intervals and normalising it to t_0.

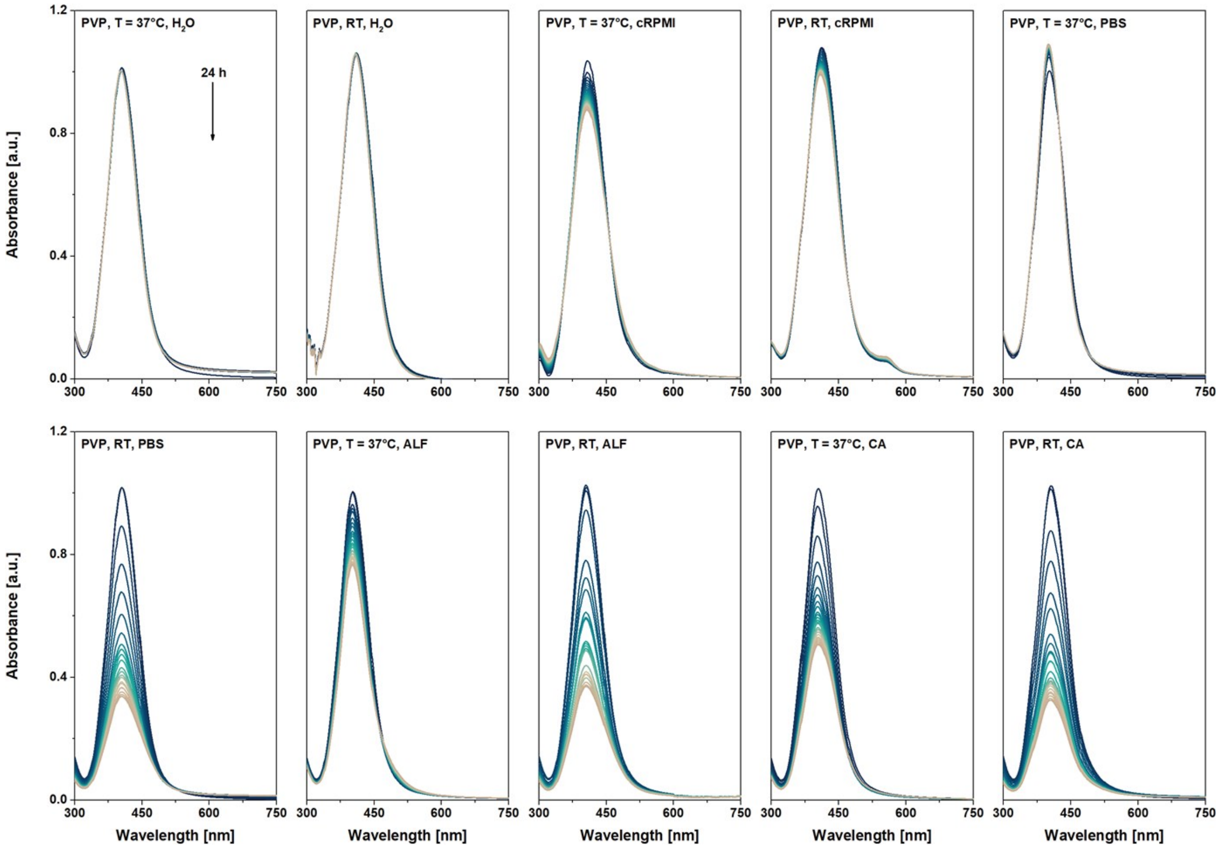

Figure S4. UV-Vis absorbance spectra of PVP surface-functionalized 30 nm AgNPs dispersed in the investigated media at 37 °C and RT. Spectra were recorded over 24 h in one-hour steps.

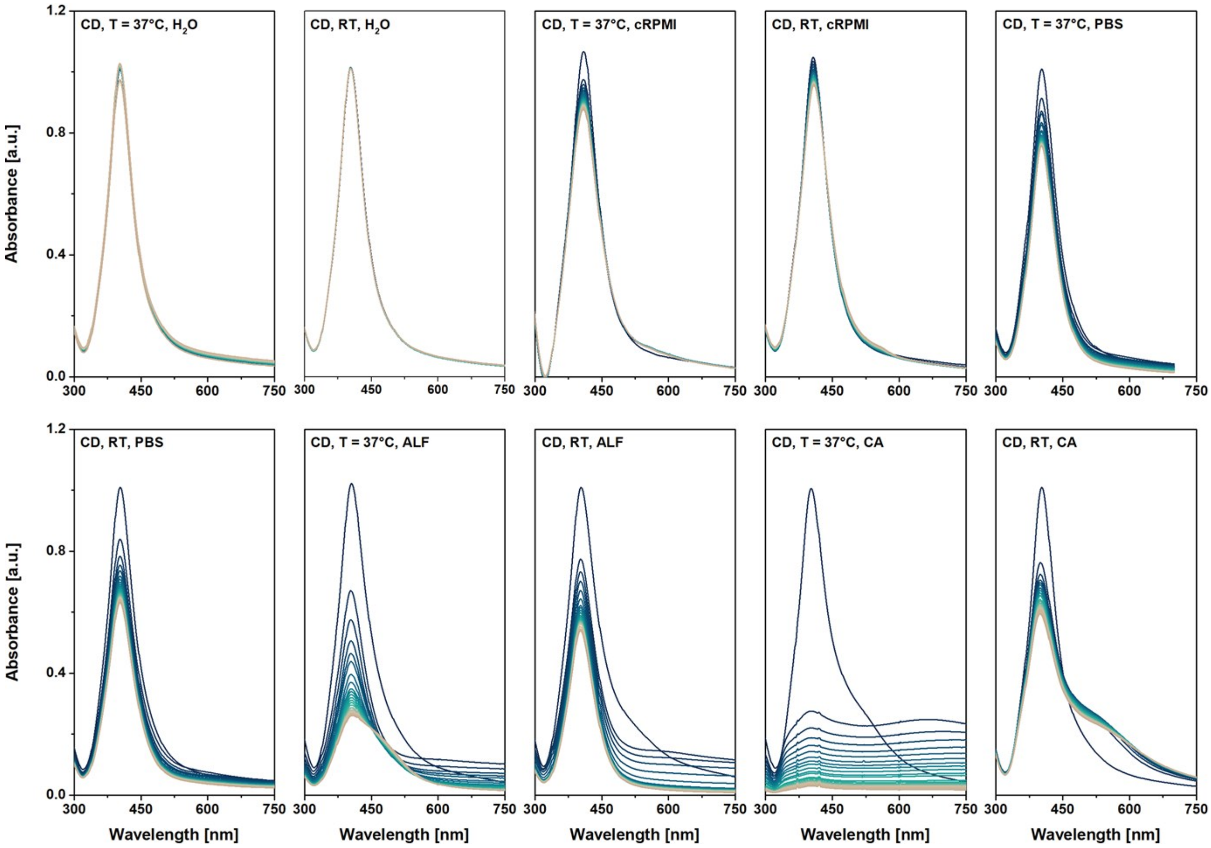

Figure S5. UV-Vis absorbance spectra of β-CD/PVP surface-functionalized 30 nm AgNPs dispersed in the investigated media at 37 °C and RT. Spectra were recorded over 24 h in one-hour steps.

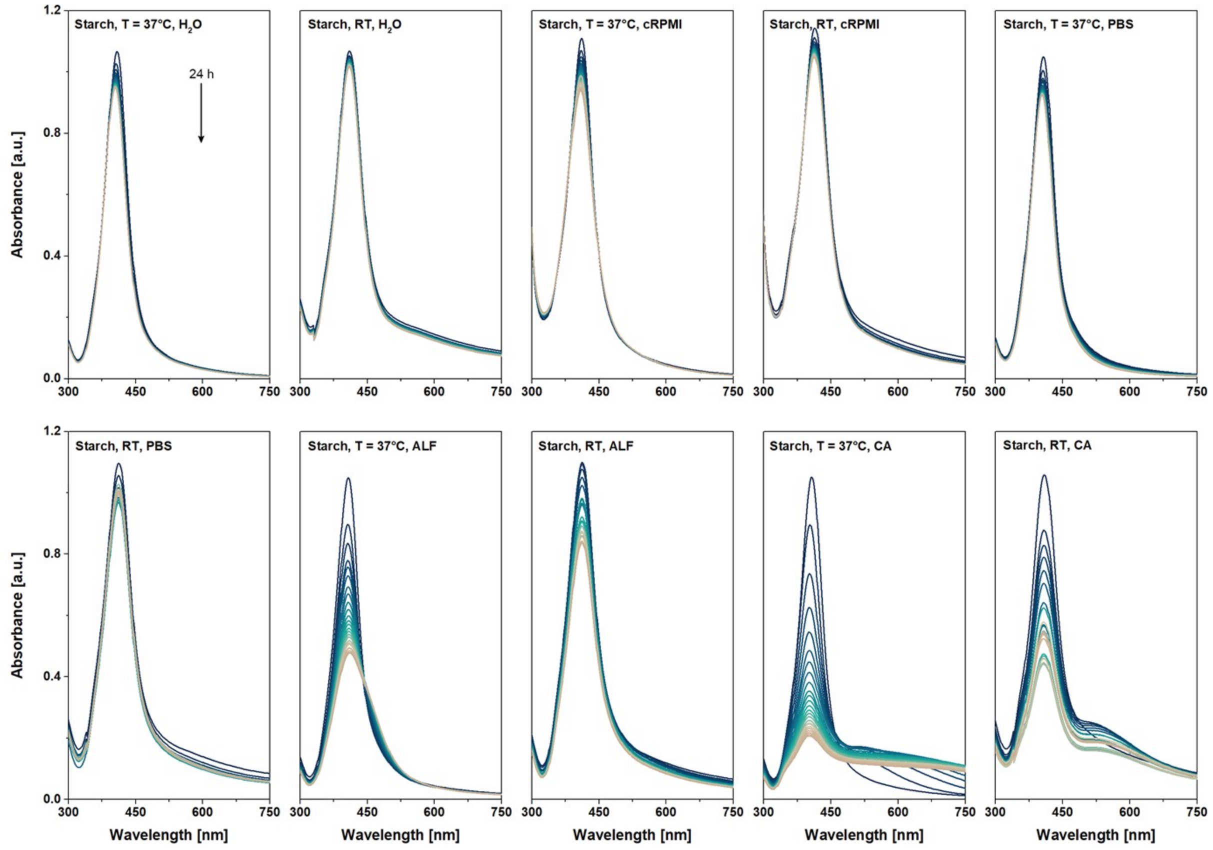

Figure S6. UV-Vis absorbance spectra of starch/PVP surface-functionalized 30 nm AgNPs dispersed in the investigated media at 37 °C and RT. Spectra were recorded over 24 h in one-hour steps.

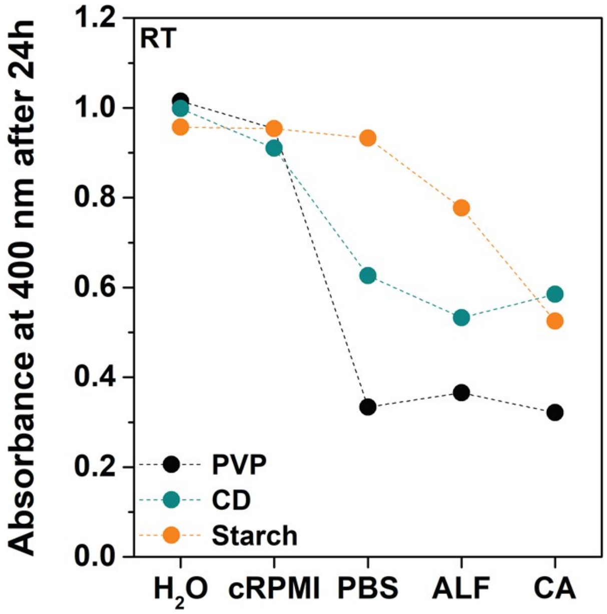

Figure S7. Dissolution of AgNPs coated with PVP only (black dots), β-CD/PVP (CD, green dots), and starch/PVP (starch, orange dots) in water, cRPMI, PBS1x, ALF1x and 0.1 M CA at 37 °C at RT after 24 h of incubation, as recorded by UV-Vis. Values were subtracted from the UV-Vis trends normalized to t_0.

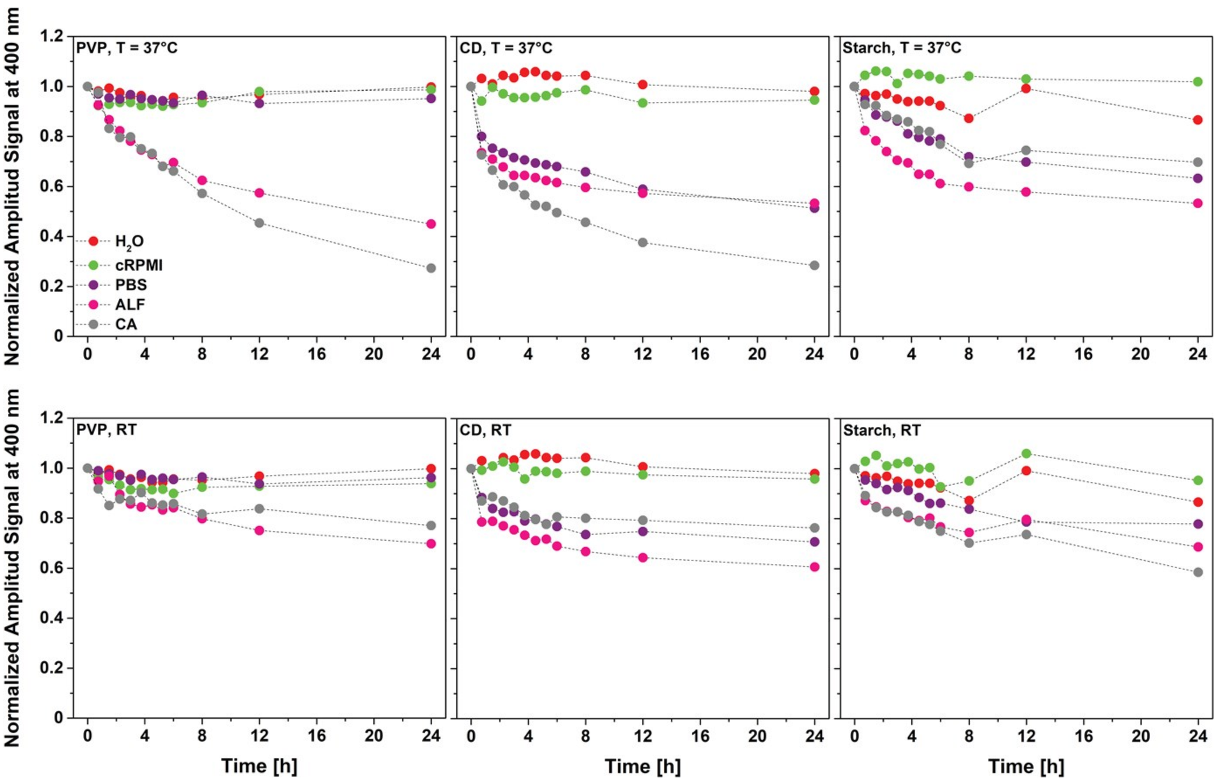

Figure S8. LIT dissolution trends of 30 nm AgNPs coated with PVP only, β-CD/PVP (CD) and starch/PVP (starch) measured over 24 h at 37 °C and RT, dispersed in water (red dots), cRPMI (green dots), PBS1x (purple dots), ALF1x (pink dots) and citric acid (CA) (grey dots). LIT amplitude signals were normalized to t_0 of every individual condition. Time points were measured every 45 min up to 6 h and then subsequently after 8, 12 and 24 h. Samples were irradiated with a light source centred at 400 nm.

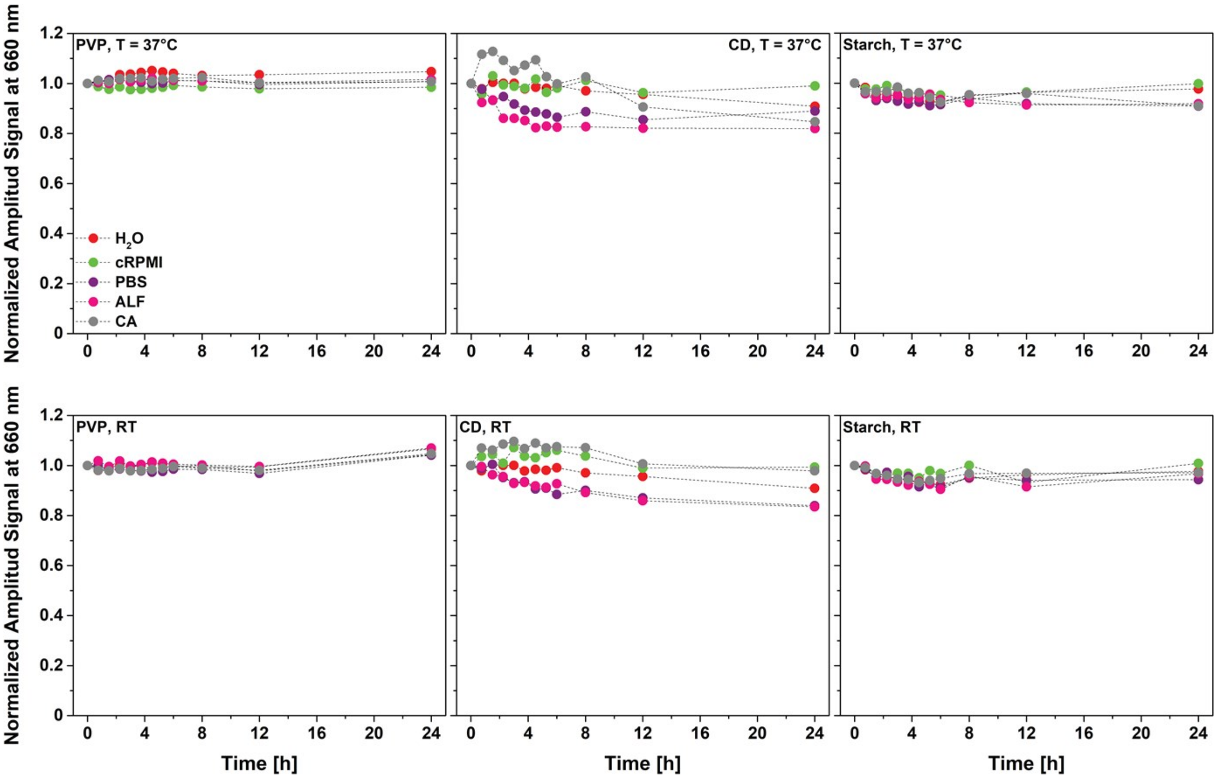

Figure S9. LIT dissolution trends of 30 nm AgNPs coated with PVP only, β-CD/PVP (CD) and starch/PVP (starch) measured over 24 h at 37 °C and RT, dispersed in water (red dots), cRPMI (green dots), PBS1x (purple dots), ALF1x (pink dots) and citric acid (CA) (grey dots). LIT amplitude signals were normalized to t_0 of every individual condition. An increase in amplitude signal corresponds to detected heat due to NP aggregation. Time points were measured every 45 min up to 6 h and then subsequently after 8, 12 and 24 h. Samples were irradiated with a light source centred at 660 nm.

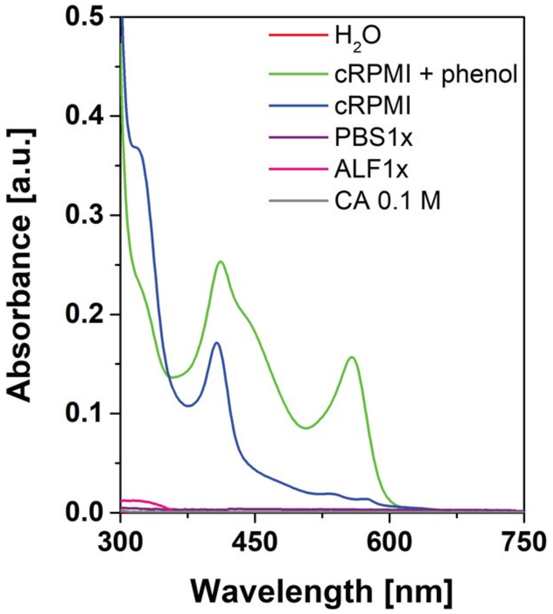

Figure S10. UV-Vis absorbance spectra of the investigated media at 37 °C. For the media only cRPMI absorbs light in the investigated wavelength range and therefore also contributes to the heat generated in LIT measurements.

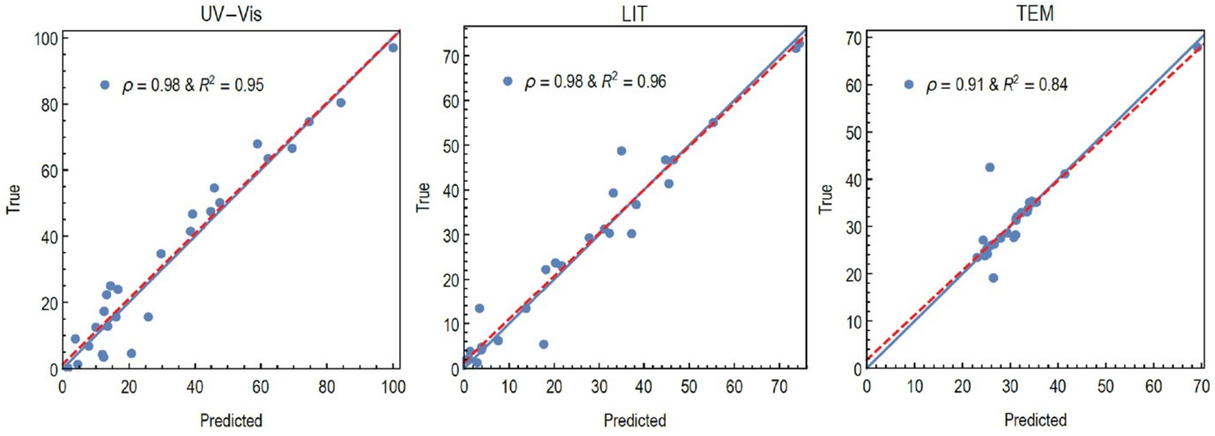

Figure S11 Supervised machine learning model based on neural networks to estimate the influence of investigated experimental conditions (i.e. applied NP coating, temperature and surrounding media). Each point represents one condition after 24 h for UV-Vis, LIT and TEM. DDLS data was excluded, as it was not possible to measure all samples until time point 24 h. The degree of goodness was measured by the Spearman's rank correlation coefficient (ρ, blue line) and is fitted against a linear regression curve (R^2, red dashed line). The model was set up using Mathematica (Version 12.0, Wolfram Language, Wolfram Research, Inc., Champaign, IL, USA).

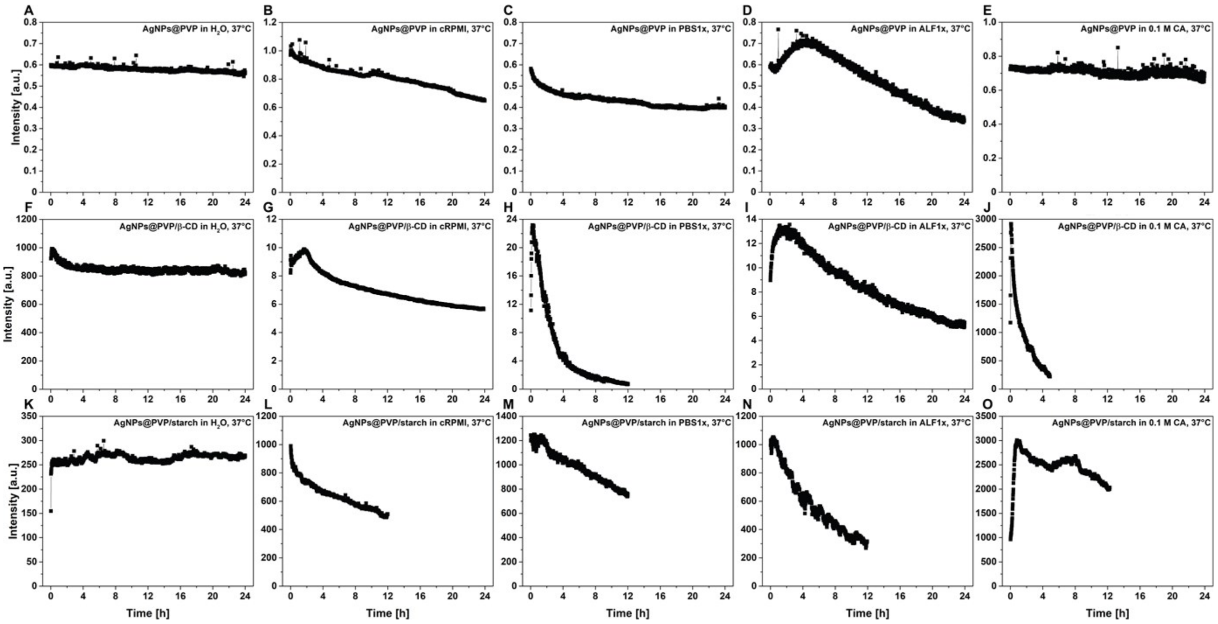

Figure S12. DDLS scattering intensity of surface-functionalized 30 nm AgNPs dispersed in the investigated media at 37 °C. Intensities were recorded over 24 h in 60 s intervals. Specific conditions (H, J, L, M, N, O) were recorded for less than 24 h, due to oversaturation of the detector caused by spikes in scattering intensity. Such spikes originate from extensive NP aggregates.

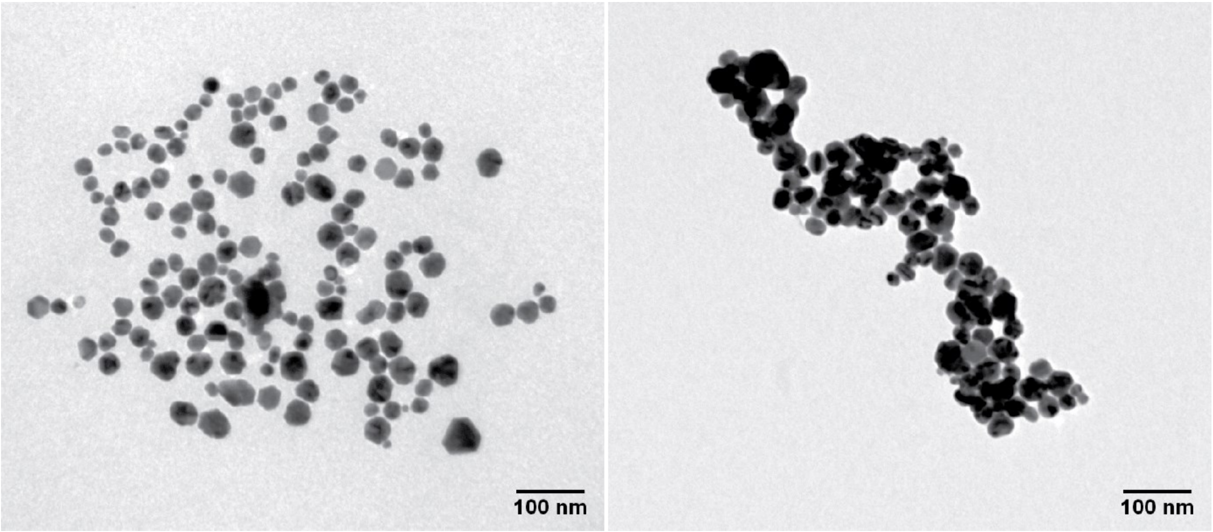

Figure S13. TEM micrographs of AgNPs@starch/PVP after 24 h at RT in Milli-Q water (left) and 0.1 M CA (right). Evaluation of NP diameter is straightforward if NPs are separated (left), but becomes non-trivial when NPs are aggregated or overlapping (right).

Table S1. Outcome of the machine learning model (Figure S11) for UV-Vis, LIT and TEM data. Two conditions were paired to predict the influence of all experimental conditions on AgNP dissolution. A higher value displays a greater significance on dissolution.

	Paired conditions					
Technique	Coating	Temperature	Coating	Medium	Temperature	Medium
UV-Vis	0.114		0.830		0.815	
LIT	0.083		0.848		0.801	
TEM	0.282		0.920		0.531	

Table S2. Core diameters of surface-functionalized AgNPs obtained by TEM. NPs were dispersed in the investigated media for 24 h.

	PVP		β-CD/PVP		starch/PVP	
[nm]	37 °C	RT	37 °C	RT	37 °C	RT
H_2O – t_0	-	31.2 ± 4	-	27.9 ± 8	-	28.8 ± 9
H_2O – t_{24h}	32.0 ± 4	31.7 ± 4	27.1 ± 8	25.2 ± 9	26.0 ± 8	27.5 ± 8
cRPMI – t_{24h}	35.3 ± 7	35.0 ± 7	26.3 ± 8	27.6 ± 7	28.2 ± 6	27.6 ± 9
PBS1x – t_{24h}	32.9 ± 6	35.1 ± 7	19.1 ± 9	23.8 ± 11	24.7 ± 5	28.6 ± 10
ALF1x – t_{24h}	33.7 ± 5	31.3 ± 7	25.4 ± 7	24.2 ± 7	23.4 ± 8	24.6 ± 9
CA 0.1 M – t_{24h}	41.1 ± 16	35.1 ± 9	67.9 ± 30	33.1 ± 8	42.5 ± 16	31.5 ± 12
